# Delay in diagnosis of generalized miliary tuberculosis with osseo-articular involvement: a case report

**DOI:** 10.1186/1752-1947-5-512

**Published:** 2011-10-10

**Authors:** Chaturaka Rodrigo, Inoshi Atukorala

**Affiliations:** 1 Department of Clinical Medicine, Faculty of Medicine, University of Colombo, Sri Lanka

## Abstract

**Introduction:**

Diagnosis of atypical tuberculosis is difficult. Therefore, it is important that physicians are aware of rare presentations of tuberculosis to avoid diagnostic delays.

**Case presentation:**

We present the case of a 17-year-old Sri Lankan man who presented to our facility with an ill-defined large induration over the skin of his left buttock and thigh. A cause could not be found despite extensive investigations. He also complained of chronic knee pain, but this was not investigated further at the time due to spontaneous resolution. Three years later his knee disease flared up again, with pain, swelling and restriction of movement. A synovial biopsy was suggestive of tuberculosis. He was started on antituberculosis therapy, to which he responded well. Our patient was asymptomatic two months after completion of therapy without any subsequent flare-ups. A chest roentgenogram taken on his second presentation showed evidence of tuberculosis sequelae in his lungs. The most likely diagnosis for the buttock and thigh swelling, when considering the entire clinical picture, is a tuberculous abscess. The constellation of skin and skeletal symptoms and pulmonary tuberculosis is a rare occurrence in an immunocompetent individual, but cases have been reported.

**Conclusions:**

This case demonstrates the different presentations and the diagnostic difficulties posed by atypical manifestations of tuberculosis. It also demonstrates the value of maintaining a high degree of suspicion in endemic areas, even in the absence of microbiological evidence.

## Introduction

The incidence of tuberculosis (TB) was assumed to be increasing in parallel with the HIV epidemic. However, latest data show that it is in fact falling slowly [1]. Once thought to be a disease of the poor and the malnourished, TB in the modern-day setting can occur anywhere regardless of socioeconomic status. Physicians need to be aware of the widely different manifestations of TB, which is a multi-system disorder. A diagnosis of atypical disease can be difficult to make and years may pass before a firm diagnosis is settled on. We present a case of tuberculosis in a young man where the atypical nature of the illness delayed the correct diagnosis for four years.

## Case presentation

A previously healthy 17-year-old Sri Lankan man first presented to the dermatology clinic of our hospital five years ago with a three-month history of a painful induration over his left buttock and hip area. Gradually, it extended onto his upper thigh with hyperpigmentation of the overlying skin, which became dry and scaly. Apart from an intermittent fever, there were no other systemic symptoms. His cardiovascular, respiratory, abdominal and nervous systems were normal on examination. Movements of his left hip were restricted in all directions. He also complained of pain and restriction of movements in the ipsilateral knee joint, which continued for two to three months before resolving spontaneously.

He was extensively investigated regarding the lump and his fever. Tuberculosis was one of the differential diagnoses considered at that time. His erythrocyte sedimentation rate (ESR) was 45 mm/hour and the results of a tuberculin skin test were negative. A blood film examination for malaria parasites, serology for typhoid/paratyphoid antigens, HIV screening and anti-nuclear antibody testing results were all negative. Results of an ultrasound of the abdomen and an echocardiogram were also normal. Skin biopsy results from the induration were negative for tuberculosis culture and detection of genomic material (TB) by polymerase chain reaction (PCR). Histology of the specimen showed a dense perivascular lymphocytic infiltrate extending into the vessel walls. There was no fibrinoid necrosis. A biopsy from the lump wall showed necrotic material.

The diagnosis was hence inconclusive. Over the next two months, his pain and fever settled spontaneously. He was managed symptomatically with antipyretics, analgesics and short courses of various antibiotic combinations. The lesion did not expand further and our patient accepted his disfigurement.

Three years later, he developed chronic pain in his right knee that was slowly progressive over four months. A diagnosis of monoarthritis was made and he was again referred to our clinic. He had a mild loss of appetite with weight loss, but no other systemic symptoms such as fever.

On examination, his right knee was swollen and tender. His movements were restricted in all directions and an effusion was palpable. The rest of the physical examination was normal.

**Figure 1:**
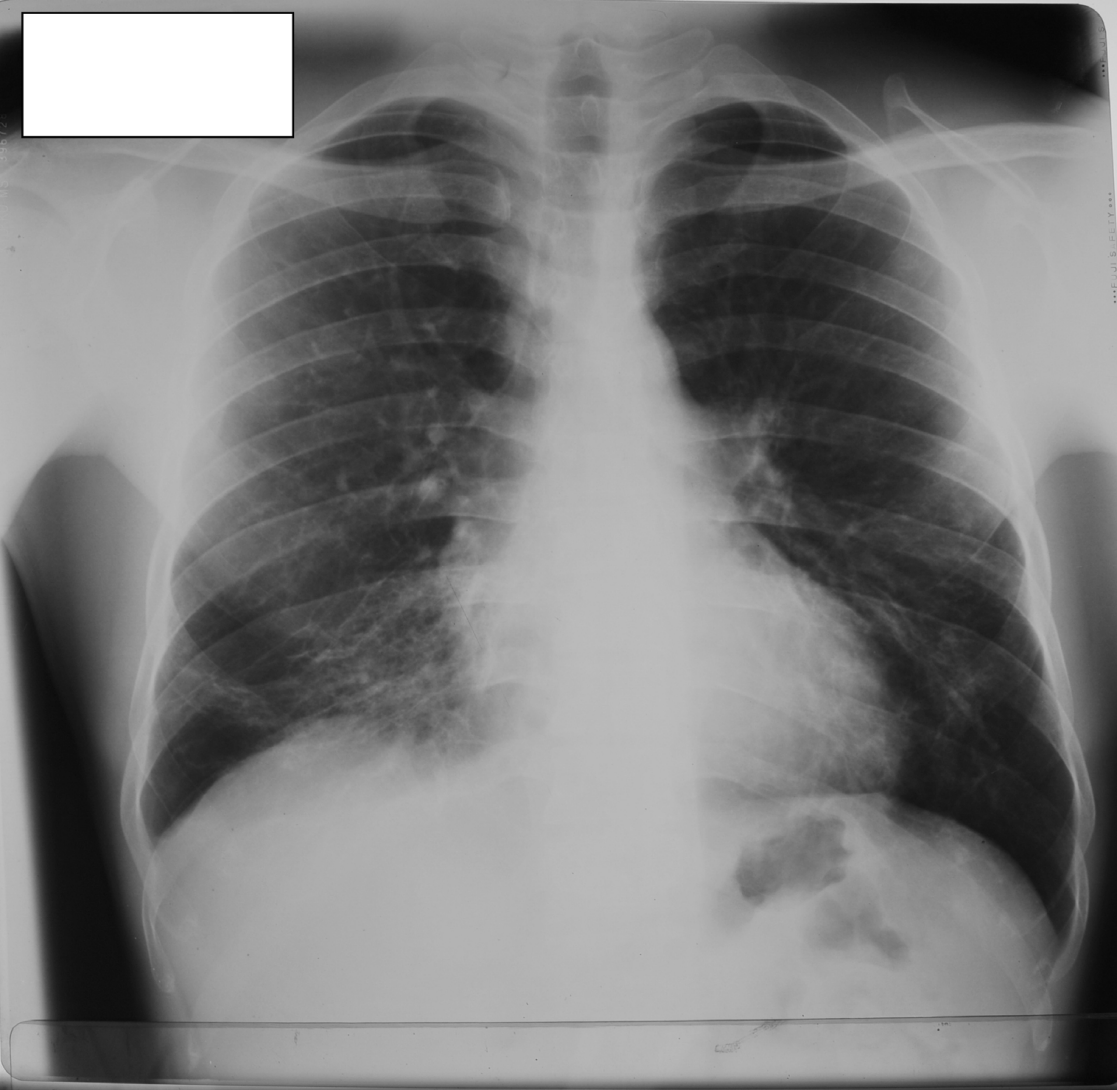
**Chest roentgenogram from our patient**. Features of previous pulmonary tuberculosis can be seen.

**Figure 2:**
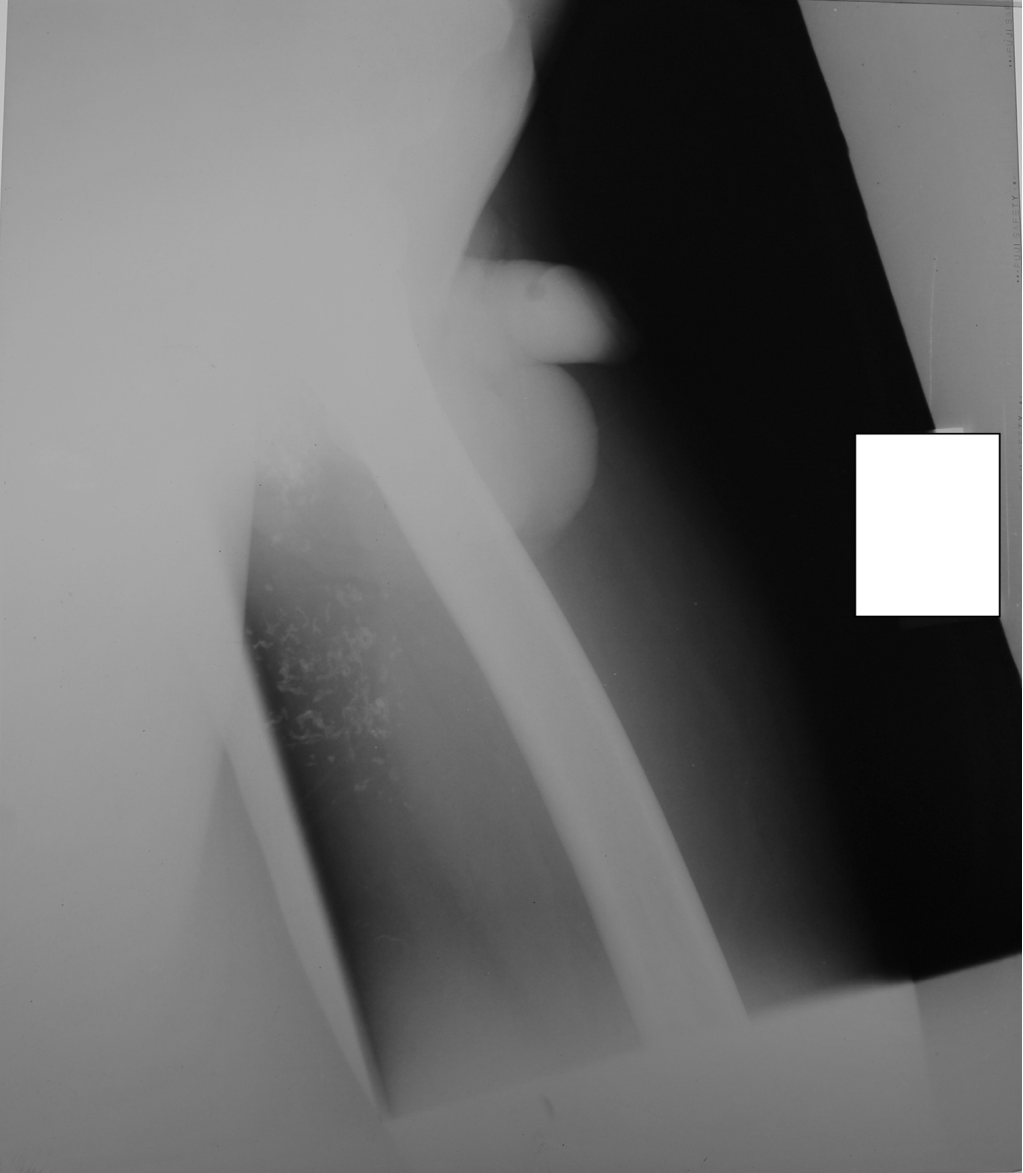
**Roentgenogram of the left hip and thigh of our patient**. Calcifications that might be the sequelae of a healed tuberculous abscess can be seen.

His basic biochemical investigations and the hematological parameters were within reference ranges apart from the ESR, which was was 55 mm in the first hour. A chest roentgenogram showed bilateral lower zone pulmonary fibrosis. There was honeycombing of the right middle lobe with traction broncheictasis plus a few calcified lymph nodes suggestive of tuberculosis sequelae (Figure 1). A roentgenogram of the left hip and thigh showed multiple calcifications, which it was hypothesized could be the remnants of a tuberculous abscess (Figure 2). The effusion of the knee joint was aspirated but it kept recurring. The appearance of the aspirate was yellow and cloudy. Biochemical analysis of the aspirate showed a protein level of 50 mg/dL, glucose level of 83.5 mg/dL and lactate dehydrogenase concentration of 2893 IU/L. Acid-fast bacilli (AFB) were not seen on direct smear. Cytological analysis revealed a leukocyte count of 6.1 × 10^9 ^cells/L (lymphocytes 70%, neutrophils 30%). Histology of the synovial biopsy showed several granuloma composed of epithelioid histiocytes located below the synovial membrane. Additionally, there were several lymphoid follicles and scattered collections of lymphocytes, plus plasma cells below the synovial membrane. This was suggestive of TB.

Anti-TB therapy was started immediately and continued for six months (isoniazid, rifampicin, pyrazinamide and ethambutol combination for two months plus isoniazid, rifampicin combination for the remainder). He was treated as an out-patient for the whole duration of his treatment. His knee pain and effusion settled with treatment and full range of movement was regained at the end of the treatment. The skin induration remained, but the underlying area hardened with anti-TB therapy. Subsequently, he was discharged from our clinic. Our patient remained symptom-free on follow-up two months after completion of treatment with no subsequent flare-ups.

## Discussion

Tuberculosis is one of the most ancient infectious diseases recorded in human history. However, its atypical presentations still elude physicians even in this era of advanced medical technology. In our patient, the diagnostic delay between his initial presentation and the initiation of anti-TB therapy was over four years.

Our patient had tuberculosis involving three different areas of the body, namely the skin, knee and the lungs. Involvement of the musculoskeletal system in tuberculosis in seen in 1% to 3% of cases and the most common sites to harbor the infection are the vertebra, hip and the knee joints [2]. The incidence of pulmonary TB with concurrent skin or skeletal TB is in the range of 50% to 65% of infections [3].

Looking at the entire clinical picture, the most likely retrospective diagnosis for our patient's lump in the buttock is a tuberculous abscess. Such abscesses involving both skin and skeletal muscle of immunocompetent individuals are rare, but have been reported [4,5]. They present with pain and swelling and follow a prolonged clinical course if diagnosis is delayed. The primary source of the bacterium can be bone, tendon sheaths, joints, direct inoculation or hematological spread (rarely). There was no history of trauma at the site of the abscess to suggest direct inoculation in our patient. It is possible that an initial pulmonary infection resulted in miliary multisystem tuberculosis via hematogenous spread. The histological features of large areas of necrosis and lymphocytic infiltration has been reported previously from tuberculous abscesses [3]. Histological features typical of granuloma or evidence of AFB are not always detected in biopsies from such abscesses. The sensitivity of TB PCR in the diagnosis of tuberculosis in skin specimens is not well established. While the sensitivity and specificity is high in patients who are immunocompromised with multibacillary skin lesions (AFB positive result from biopsies), the sensitivity in paucibacillary immunocompetent individuals is in the range of 55% to 73% [6]. This might explain the initial negative results from the skin biopsy when tested with TB PCR.

Regarding tuberculosis of the knee, it can follow an indolent course and become reactivated years later [7]. At the time of appearance of the buttock lump, our patient also complained of knee pain, which might have been due to TB monoarthritis. Several similar case reports of TB monoarthritis of the knee leading to the diagnosis of multisystem tuberculosis (after much delay) have been reported in literature. In many of these instances, the initial respiratory symptoms were overlooked [8-12]. Though AFB were never identified in the synovial biopsy or culture, the histological evidence and other circumstantial evidence including the complete resolution of symptoms of the knee with anti-TB therapy support a diagnosis of tuberculosis in our patient.

The treatment of skeletal tuberculosis can be initiated with anti-TB chemotherapy alone or with a combination of chemotherapy and surgery. In arthritis of knee joint of adults, early synovectomy and joint debridement followed by anti-TB chemotherapy for six to 12 months is recommended. In severe destructive joint disease, arthrodesis is the preferred mode of treatment. Tuberculosis involving other skeletal structures such as spine and hip may require extensive chemotherapy over one to two years [13]. Earlier recommendations were to manage the innocuous tuberculous skin abscesses non-surgically. However, the current thinking is that surgical debridement, wide resection of involved bones, cartilages and soft tissue with reconstruction gives better results when combined with anti-TB chemotherapy [14]. Collections in deep tissues (for example, paraspinal and iliopsoas collections) require percutaneous computed tomography (CT)-guided drainage [15].

## Conclusions

Tuberculosis is still a diagnostic challenge, especially when the presentation is atypical and extra-pulmonary. Unless a high degree of suspicion is maintained, the diagnosis can be missed for years at great cost to patients and the system. In endemic areas it may be justifiable to treat for tuberculosis empirically without microbiological evidence when the clinical, histological and other circumstantial evidence favor it.

## Consent

Written informed consent was obtained from the patient for publication of this case report and any accompanying images. A copy of the written consent is available for review by the Editor-in-Chief of this journal.

## Competing interests

The authors declare that they have no competing interests.

## Authors' contributions

All authors participated in the design, literature search, information coding and writing of the manuscript. All authors read and approved the final manuscript
